# Inhibition of Mild Steel Corrosion in Hydrochloric Acid Solution by New Coumarin

**DOI:** 10.3390/ma7064335

**Published:** 2014-06-05

**Authors:** Abdul Amir H. Kadhum, Abu Bakar Mohamad, Leiqaa A. Hammed, Ahmed A. Al-Amiery, Ng Hooi San, Ahmed Y. Musa

**Affiliations:** 1 Department of Chemical and Process Engineering, Faculty of Engineering and Built Environment, Universiti Kebangsaan Malaysia, Bangi, Selangor 43600, Malaysia; E-Mails: amir@eng.ukm.my (A.A.H.K.); drab@eng.ukm.my (A.B.M.); hooisan_ng@hotmail.com (N.H.S.); 2 Environmental Research Center, University of Technology, Baghdad 10066, Iraq; E-Mail: leiqaa.technology@gmail.com (L.A.H.); 3 Department of Chemistry, Western University, London, ON N6A 5B7, Canada; E-Mail: amusa6@uwo.ca

**Keywords:** corrosion inhibitor, coumarin, potentiodynamic, terephthalaldehyde

## Abstract

A new coumarin derivative, *N*,*N*′-((2E,2′E)-2,2′-(1,4-phenylenebis(methanylylidene))bis(hydrazinecarbonothioyl))bis(2-oxo-2H-chromene-3-carboxamide) PMBH, was synthesized and its chemical structure was elucidated and confirmed using spectroscopic techniques (Infrared spectroscopy IR, Proton nuclear magnetic resonance, ^1^H-NMR and carbon-13 nuclear magnetic resonance ^13^C-NMR). The corrosion inhibition effect of PMBH on mild steel in 1.0 M HCl was investigated using corrosion potential (*E*_CORR_), potentiodynamic polarization, electrochemical impedance spectroscopy (EIS), and electrochemical frequency modulation (EFM) measurements. The obtained results indicated that PMBH has promising inhibitive effects on the corrosion of mild steel in 1.0 M HCl across all of the conditions examined. Scanning electron microscopy (SEM) was used to investigate the morphology of the mild steel before and after immersion in 1.0 M HCl solution containing 0.5 mM of PMBH. Surface analysis revealed improvement of corrosion resistance in presence of PMBH.

## 1. Introduction

Studies on preventing the corrosion of steel in acidic environments and the problematic chemical processes that arise have attracted the attention of researchers from a wide range of industrial sectors [1]. Corrosion is a common problem for steel and directly impacts its cost and safety. The corrosion of iron, or rust, can cause structural damage and lead to changes in the mechanical and chemical properties of plants, vessels, pipes, and other processing equipment. These effects demonstrate that corrosion would produce considerable costs if an effective solution is not identified from its study and research. Preventing the corrosion of steel has played an important role in various industries, especially in the chemical and petrochemical processing industries that employ the use of steel. A number of studies have been conducted to investigate effective methods for preventing corrosion. Acids are widely used in industrial processes, such as pickling, cleaning, descaling, *etc*. Inhibitors are effective in reducing the dissolution rate of metals [2,3,4,5,6]. The primary step in the action of inhibitors in an acidic solution is adsorption onto the metal surface, which is usually free of oxides. The adsorbed inhibitor then acts to retard the cathodic and or anodic electrochemical corrosion reaction. It is often not possible to assign a single general mechanism to an inhibitor because the mechanism may change with the experimental conditions. Therefore, the inhibition mechanism of an inhibitor may vary with several factors, such as the concentration, pH, nature of the anion of the acid and nature of the metal. The mechanisms of action of inhibitors that possess the same functional group may also vary with several factors, such as the effect of the molecular structure on the electron density of the functional group and the size of the aromatic and aliphatic protons of the molecule [7,8,9]. Corrosion inhibitors are of considerable practical importance because they are extensively employed in both reducing metallic wastes during production and minimizing the risk of material failure (and the consequent sudden shut-down in industrial processes that leads to added costs) [10]. It is important to use corrosion inhibitors to prevent metal dissolution and minimize acid consumption [11,12,13]. The majority of well-known acid inhibitors are organic compounds that contain nitrogen, sulfur and oxygen atoms. The inhibitory action exercised by organic compounds on the dissolution of metallic species is normally related to adsorption interactions between the inhibitors and the metal surface. This process is considered to represent an interface inhibition according to Fischer’s classification. The surfactant inhibitor has many advantages, such as a high inhibition efficiency, low price, low toxicity, and easy production [14,15,16]. The problem of finding an inhibitor that has little or no impact on the environment has recently motivated numerous studies [17]. Chemical inhibitors are often used for these processes, primarily to control the metal dissolution and acid consumption. The majority of the well-known acid corrosion inhibitors are organic compounds that contain nitrogen, sulfur or oxygen atoms [18,19]. The planarity (*p*) and lone pairs of electrons present on N, O and S atoms are the important structural features that control the adsorption of these molecules onto the surface of the metal. The importance of this work lies in verifying the already established results on the corrosion inhibition effect of various Schiff bases on mild steel in acidic media [20].

Despite the great number of studies devoted to the subject of corrosion inhibitors most of what is known is as a result of trial and error, both in the laboratory and the fields. Historically, the development of corrosion inhibitors has always been determined by their effectiveness, and they were often based on ecologically problematic heavy metal. The development of new corrosion inhibitors of non-toxic type, which do not contain heavy metals and inorganic phosphates, is of considerable importance [21]. Inorganic compounds such as chromate, dichromate, nitrite, and nitrate are widely used as corrosion inhibitors in several media and for different metals and alloys, on the other hand, the biotoxicity of these products, especially chromate, is well documented, as well as their non-environmental-friendly characteristics which limit their application [22]. Among alternative corrosion inhibitors, organic products containing one or more polar functions have proven to be quite efficient in minimizing the effect of corrosion in addition to heterocyclic compounds containing polar groups and π-electrons [23]. Effective inhibitors are expected to perform under a wide range of conditions; hence, special attention must be paid to the selection of inhibitors for such practical applications [24].

In this study, a new coumarin derivative, PMBH, was synthesized and its chemical structure was elucidated and confirmed using spectroscopic techniques. The inhibitory effect of PMBH was investigated on the corrosion of mild steel in 1.0 M HCl using various electrochemical measurements. Surface analyses were performed on the corroded surfaced using scanning electronic microscopy (SEM).

## 2. Results and Discussion

### 2.1. Synthesis

**Scheme 1:**
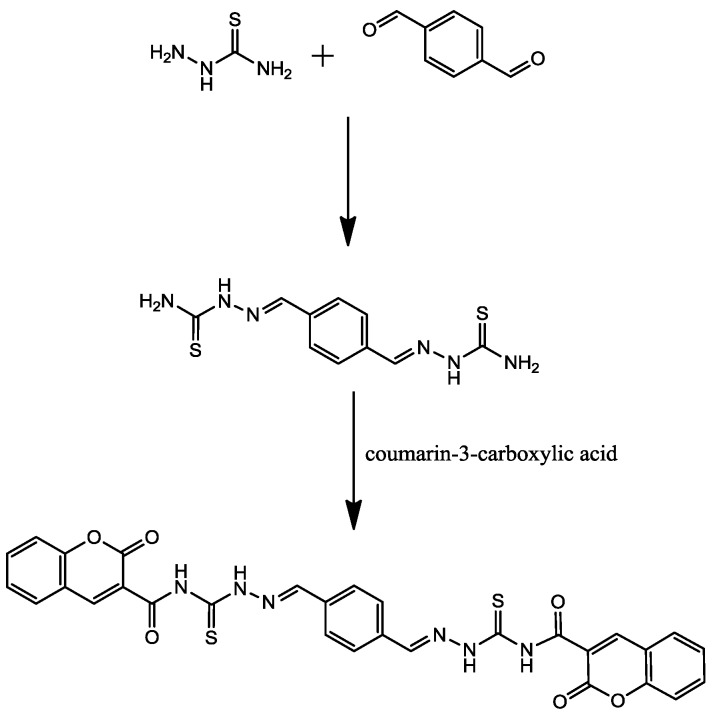
The chemical synthesis of PMBH.

To synthesize the new corrosion inhibitor PMBH, the reaction sequence outlined in Scheme 1 was followed, starting from commercially available thiosemicarbazide. The synthesis was carried out by refluxing thiosemicarbazide with terephthalaldehyde in the presence of a few drops of hydrochloric acid, followed by reaction with coumarin-3-carboxylic acid.

(^1^H-NMR) (CDCl_3_): δ 7.617 (s, N=C–H), δ 7.630 (s, N–H), δ 8.572 (s, 2H, NH), δ 8.953 (s, 1H, C=C–H) 7.270, 7.333, 7.346, 7.358, 7.372, 7.477, 7.492, 7.507 (s, 2H, coumarin aromatic ring), 7.672, 7.768, 7.782, 7.796 (s, 1H, aromatic ring); ^13^C-NMR (CDCl_3_): 116.82, 117.88, 118.00, 124.90, 126.25, 129.57, 134.47, 149.11, 155.25, 156.69, 163.73; FT-IR: 3400.2, 3268.0, 3197.2 cm^−1^ (N–H), 3017.0 cm^−1^ (C–H, aromatic), 1710.5 cm^−1^ (C=O, coumarin), 1600.0 cm^−1^ (C=O, amide); 1590.1 cm^−1^ (C=C, alkene), 1518.8 cm^−1^ (C=N, imine), 1500.5 cm^−1^ (C–C, aromatic), 1285.3, and 1082.7 cm^−1^ (C–O–C, sym and asym).

### 2.2. Electrochemical Measurements

#### 2.2.1. Corrosion Potential (*E*_CORR_) Measurements

**Figure 1:**
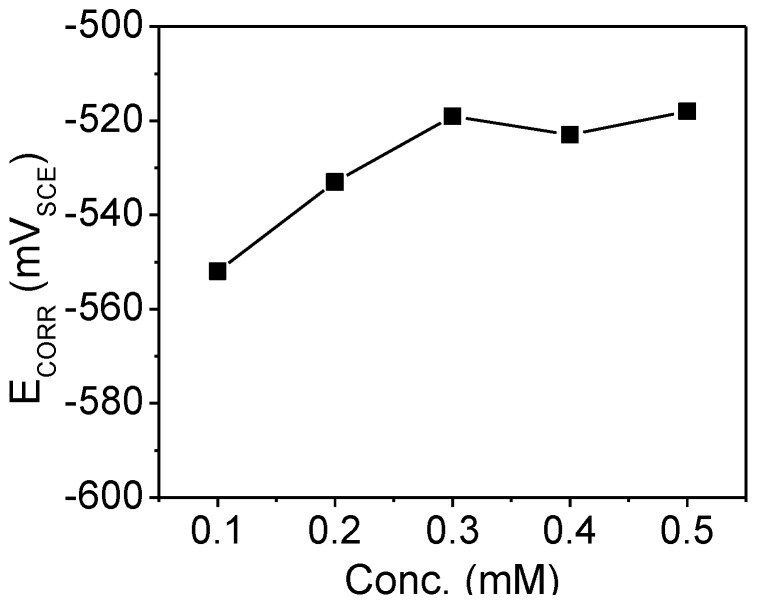
*E*_CORR_ values for mild steel in a HCl solution with various concentrations of PMBH at 30 °C.

**Figure 2:**
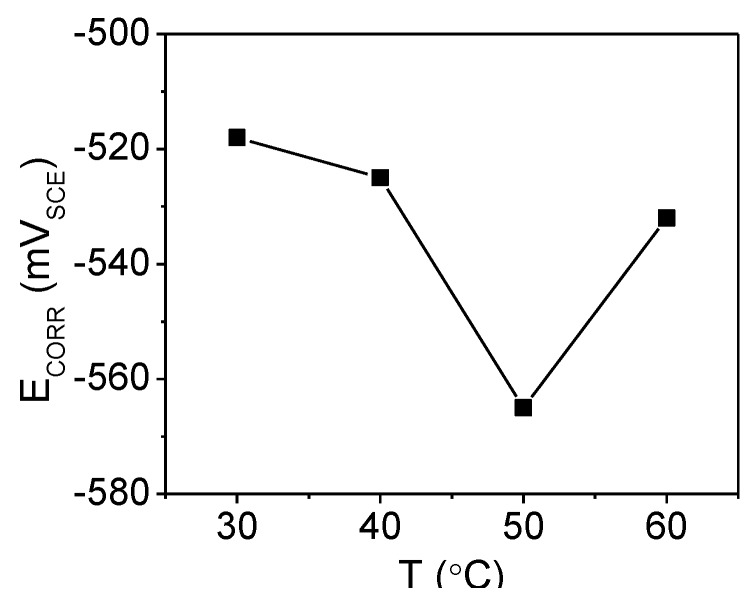
*E*_CORR_ values for mild steel in a HCl solution with 0.5 mM of PMBH at various solution temperatures.

Changes in the *E*_CORR_ values in the presence of an inhibitor are often useful indicators of which reaction, cathodic or anodic, is more affected [25]. The *E*_CORR_ of mild steel was monitored at various temperatures in different PMBH concentration. Figure 1 shows the effect of the presence of PMBH inhibitor on the variation of the *E*_CORR_ of mild steel as a function of temperature in the 1.0 M HCl solution. It is clear from Figure 1 that the addition of PMBH to the acidic solution shifts the *E*_CORR_ values toward positive direction at studied temperatures. This indicates that PMBH has the ability to inhibit the acidic corrosion of mild steel at 30 °C. The maximum shift in the *E*_CORR_ value was 35 mV at 30 °C. This preliminary result suggests that PMBH can hinder both reactions under open circuit conditions, including the oxidation of the oxide-free iron and the discharge of the hydrogen ions to produce hydrogen gas on the surface of the mild steel [25]. The effects of solution temperature on the *E*_CORR_ in inhibitor-containing solution were also investigated as shown in Figure 2. A different behavior is observed where the *E*_CORR_ value shifts toward negative direction with increasing solution temperature. The solution temperature influences the metal dissolution rate and inhibitor molecule adsorption/desorption rate. The decrease in the corrosion potential value is due to the increasing in the metal dissolution rate and the increase in desorption rate of the adsorbed inhibitor’s molecule on the metal surface. This indicates that the studied inhibitor fails in protecting the mild steel at temperature higher than 30 °C. Further investigation of how this inhibitor behaves was carried out using other electrochemical techniques.

#### 2.2.2. Polarization Measurements

**Figure 3:**
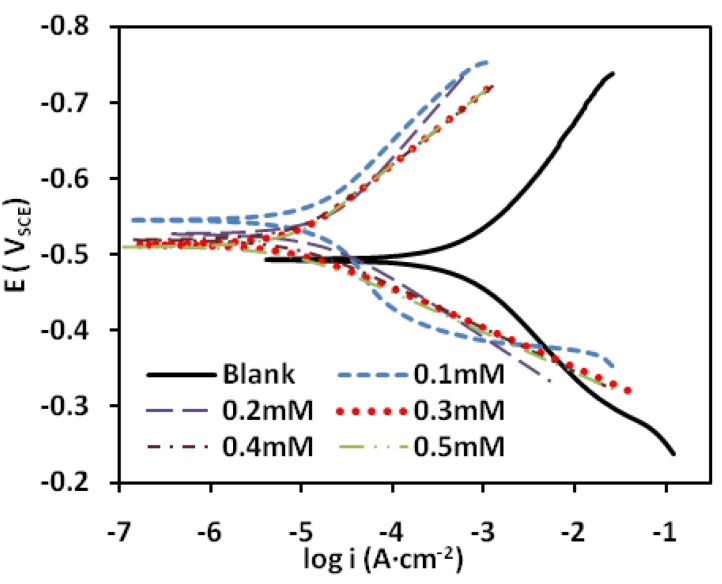
Potentiodynamic polarization curve for mild steel in 1.0 M HCl with various concentrations of the PMBH at 30 °C.

**Figure 4:**
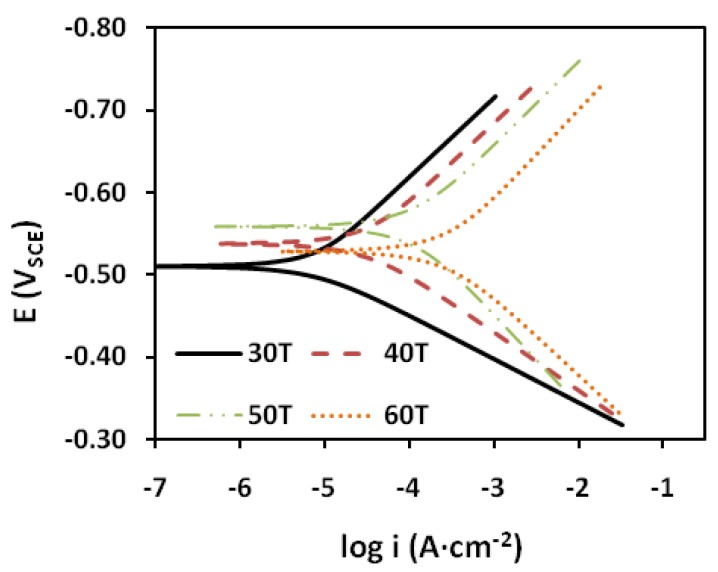
Potentiodynamic polarization curve for mild steel in 1.0 M HCl with 0.5 mM of the PMBH at various temperatures.

The polarization profile of mild steel in 1.0 M HCl is shown in Figure 3 and Figure 4. The numerical values of the variation of the corrosion current density *(i*_corr_*)*, the corrosion potential (*E*_CORR_), the anodic Tafel slope (βa) and the cathodic Tafel slope (βc)with various concentrations of the PMBH inhibitor and at various solution temperatures were obtained from polarization profiles and presented in Table 1 and Table 2.

**Table 1:** Polarization parameters for mild steel in 1.0 M HCl with different concentrations of the PMBH at 30 °C.

Conc.(mM)	*i*_corr_ (μA·cm^−2^)	*E*_CORR_ (V_SCE_)	β_a_ (V·dec^−1^)	β_c_ (V·dec^−1^)	*IE*(%)
0	660.1	−0.49	0.12	0.14	0
0.1	19.1	−0.53	0.08	0.14	97.1
0.2	16.2	−0.55	0.13	0.13	97.5
0.3	8.76	−0.52	0.06	0.09	98.6
0.4	7.72	−0.51	0.05	0.1	98.8
0.5	7.36	−0.51	0.05	0.1	98.8

**Table 2:** Polarization parameters for mild steel in 1.0 M HCl with 0.5 mM of PMBH at different temperatures. The *i*_corr_ values for the blank solution at different temperatures were taken from our previous study [26].

*T* (°C)	*i*_corr_ (μA·cm^−2)^	*E*_CORR_ (V_SCE_)	βa (V·dec^−1^)	βc (V·dec^−1^)	*IE* (%)
30	7.3	−0.51	0.05	0.10	98.8
40	35	−0.538	0.07	0.10	96.6
50	124	−0.558	0.12	0.10	94.3
60	401	−0.528	0.14	0.12	93.8

These values were calculated from the intersection of the anodic and cathodic Tafel lines of the polarization curve at *E*_CORR_. The inhibition efficiency (*IE*) was calculated using the following equation:

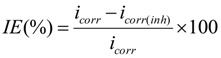
(1)
where *i*_corr_ and *i*_corr(inh)_ are the corrosion current densities in the absence and presence of the inhibitor, respectively.

Table 1 and Table 2 showed that *i*_corr_ increases with increasing solution temperature, whereas *i*_corr_ decreases with the addition of PMBH to the acidic solution throughout the investigated temperature range. This behavior can be explained as follows: the inhibitor adsorbed onto the metal surface, and an increase in temperature resulted in the desorption of some of the adsorbed inhibitor molecules, leading more metal surface exposed to the acidic medium, thus, increases the metal dissolution rate which caused a decrease in the inhibition efficiency [26]. The addition of the PMBH inhibitor caused the calculated *E*_CORR_ values to shift towards more positive values which reflect the inhibitory effect of PMBH on the corrosion of mild steel at 30 °C however this value decreases with solution temperature which indicates a decrease in the level of the protection of PMBH. Figure 3 and Figure 4 revealed that the anodic and cathodic processes changed with the addition of various concentrations of the PMBH. A compound can be classified as an anodic- or cathodic-type inhibitor when the change in *E*_CORR_ is greater than 85 mV [27]. Because the largest displacement exhibited by PMBH is 54 mV at 30 °C (Table 1), Therefore, the studied inhibitor functions as a mixed-type inhibitor, which indicates that the addition of PMBH to the acidic solution reduced the anodic dissolution of the mild steel and retarded the cathodic hydrogen.

#### 2.2.3. Electrochemical Impedance Spectroscopy (EIS) Measurements

**Figure 5:**
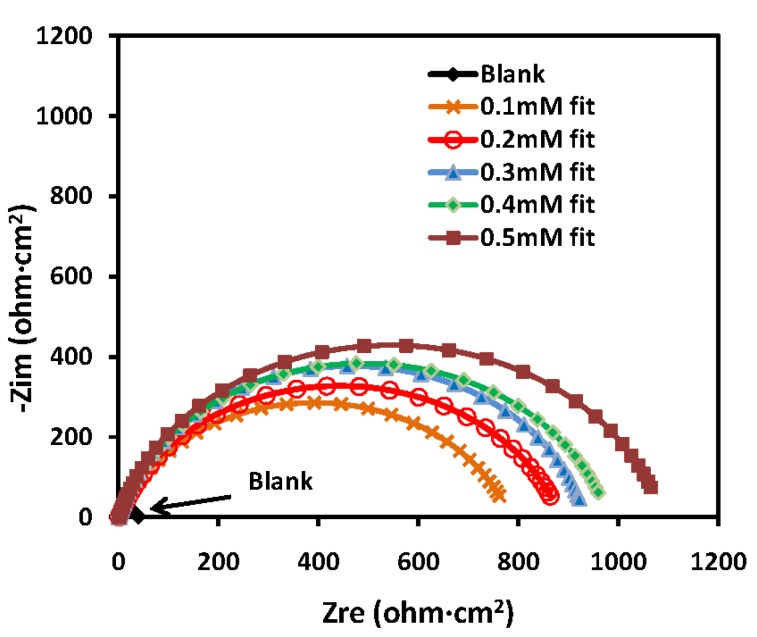
Nyquist plots for mild steel in 1.0 M HCl with various concentrations of PMBH at 30 °C.

**Figure 6:**
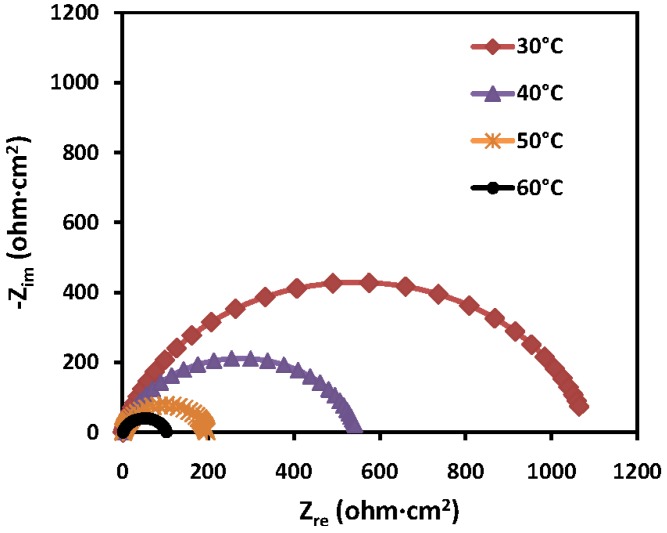
Nyquist plots for mild steel in 1.0 M HCl with 0.5 mM PMBH at various temperatures.

**Figure 7:**
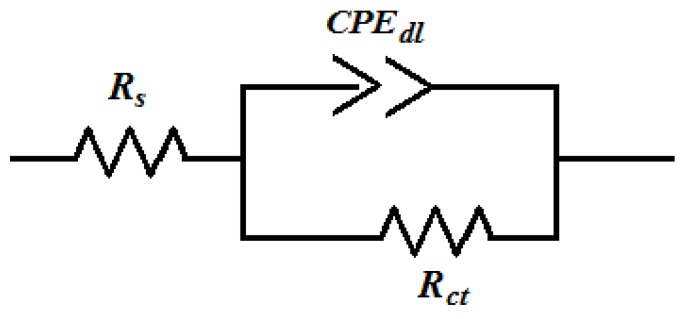
Equivalent model used to fit the impedance data for mild steel in 1.0 M HCl in the absence and the presence of PMBH.

The experimental results obtained from the EIS measurements for the corrosion of mild steel in the absence and presence of the inhibitor at 30, 40, 50 and 60 °C are summarized in Table 3 and Table 4. The impedance spectra for the mild steel samples in 1.0 M HCl in the absence of PMBH or in the presence of various concentrations of PMBH at 30 °C are presented as Nyquist plots in Figure 5. A considerable increase in the total impedance was observed with the addition of PMBH. It can be concluded from Figure 5 that the impedance response of the mild steel was significantly altered after the addition of PMBH to the corrosive solution. This result can be attributed to an increase in the substrate impedance with the increase in the concentration of the inhibitor. However the total impedance of the mild steel in the presence of 0.5 mM of PMBH decreases with increasing solution temperature, Figure 6. This behavior is due to desorption of the adsorbed inhibitor’s molecules from the mild steel surface. In the impedance spectrum of the mild steel in the presence of PMBH, the Nyquist plots have two loops: one loop in the high frequency region (HF) and one loop at an intermediate frequency (MF), with slight inductive behavior at low frequencies (LF). The HF and MF loops were attributed to the electrode and to the charge-transfer process, respectively. The inductive behavior observed in the LF region was attributed to the relaxation of the adsorption of corrosion products or to the adsorption of the inhibitor molecules on the mild steel surface in the acid solution in the absence and presence of inhibitor, respectively [28,29]. Similar behavior was observed for all of the temperatures that were examined. Since the corroding surface of the working electrode is expected to be inhomogeneous because of its roughness; therefore, the capacitance is presented through a constant phase element (CPE). The EIS results were analyzed using the equivalent circuit mentioned in [26] as shown in Figure 7.

The inhibition efficiencies (*IE*%) were calculated from the charge transfer resistance using the equation shown below:

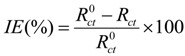
(2)
where *R*^0^_ct_ and *R*_ct_ indicate the values of the charge transfer resistances in the presence or the absence of corrosion inhibitor.

**Table 3:** Inhibition efficiency of PMBH calculated from electrochemical impedance spectroscopy (EIS) results with various concentrations at 30 °C.

Conc. (mM)	*R*_ct_ (ohm·cm^2^)	*IE* (%)
Blank	42	–
0.1	782	94.6
0.2	881	95.2
0.3	932	95.5
0.4	977	95.7
0.5	1087	96.1

**Table 4:** Inhibition efficiency of PMBH calculated from EIS results as a function of temperatures.

*T* (°C)	Conc. (mM)	*R*_ct_ (ohm·cm^2^)	*IE* (%)
30	Blank	42	–
30	0.5	1087	96.1
40	Blank	13	–
40	0.5	546	97.5
50	Blank	7	–
50	0.5	195	96.4
60	Blank	3	–
60	0.5	101	97.0

It can be seen from Table 3 and Table 4 that the *R*_ct_ values increase with increasing concentration but decreases significantly with increasing solution temperature.

#### 2.2.4. Electrochemical Frequency Modulation

Electrochemical frequency modulation (EFM) is a new electrochemical technique for determining the corrosion rate without a preliminary knowledge of the Tafel constants. The principal advantages of this technique include the measurement of the corrosion rate, the Tafel parameters and the causality factors in a single data set [30]. While using EFM, a potential perturbation signal composed of two sine waves is applied to any corroding specimen to obtain a current response. EFM has been used for different combinations of metals and electrolytes to accurately measure the corrosion parameters. This technique is similar to the harmonic method in that it employs a lower amplitude (20 mV) sinusoidal perturbation signal, but unlike the harmonic method, it is composed of two sine waves instead of one. EFM has many advantages over the harmonic method, including data validations, a larger current response and an insensitivity to harmonics in the perturbation signal. The corrosion parameters including the corrosion efficiency *IE* (%), the corrosion current density (μA·cm^−2^), the Tafel constant and the causality factors (CF-2 and CF-3) with different concentrations of PMBH in 1.0 M HCl at 30 °C and at different temperatures are listed in Table 5 and Table 6.

**Table 5:** The electrochemical frequency modulation (EFM) parameters for mild steel in 1.0 M HCl with various concentrations of the PMBH at 30 °C.

Conc. (mM)	*i*_corr_ (μA·cm^−2)^	βa (V·dec^−1^)	βc (V·dec^−1^)	C.R. (mpy)	*IE* (%)	CF-2	CF-3
Blank	81.7	0.09	0.27	90.2	–	1.97	2.02
0.1	61.0	0.05	0.06	27.5	25.3	4.56	2.49
0.2	43.7	0.16	0.17	20	46.4	2.79	2.11
0.3	22.4	0.09	0.10	10.2	72.5	1.79	1.70
0.4	20.9	0.08	0.09	9.5	74.3	1.80	1.78
0.5	17.8	0.08	0.09	8.1	78.2	1.34	2.42

**Table 6:** EFM parameters for mild steel in 1.0 M HCl with 0.5 mM PMBH at various temperatures.

*T* (°C)	*i*_corr_ (μA·cm^−2)^	β_a_ (V·dec^−1^)	β_c_ (V·dec^−1^)	C.R. (mpy)	*IE* (%)	CF-2	CF-3
30	17.8	0.08	0.09	8.1	97.4	1.34	2.42
40	37.0	0.08	0.10	16.9	96.4	2.01	4.63
50	128.1	0.08	0.16	58.6	94	1.91	2.99
60	370.6	0.09	0.15	123.7	94	1.91	5.67

As shown in Table 5, the *i*_corr_ decreases with increasing inhibitor concentration. The standard values for CF-2 and CF-3 are 2.0 and 3.0, respectively. If the causality factors differ from 2 to 3, one can conclude that the measurement is affected by noise. If the value of the causality factor approximates the standards, a correlation exists between the perturbation signal and the response signal; therefore, the data are accepted. If CF-2 and CF-3 are in the range of 0–2 and 0–3, the EFM data are valid. Any deviation in the causality factor from the theoretical value may be due to a perturbation amplitude that is too small, in insufficient resolution in the spectrum frequency, or an inhibitor that is not functioning properly [8]. As observed before with other measurements, the inhibition efficiency of PMBH increases with increasing PMBH concentration but decreases with solution temperature at a given concentration. This revealed that the inhibitor molecules adsorbed physically on the mild steel surface and not chemically therefore increasing the temperature enhances the both the dissolution of metal and the desorption of inhibitor molecules from metal surface.

### 2.3. Scanning Electron Microscopy (SEM)

**Figure 8:**
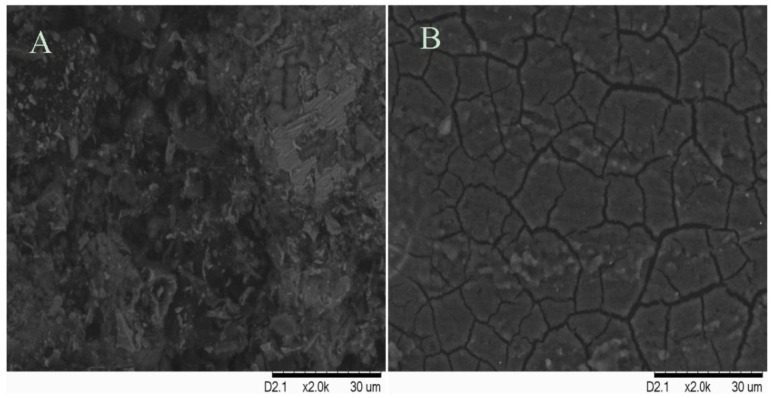
SEM micrographs of mild steel in 1.0 M HCl solution at 30 °C in the absence (**A**) and presence (**B**) of 0.5 mM PMBH.

SEM analysis was performed to investigate the surface morphology of the mild steel after immersion in 1.0 M HCl in the absence and the presence of 0.5 mM PMBH for 3 h at 30 °C, Figure 8. Damaged surface was observed in the absence of PMBH due to high dissolution rate of iron at such pH however a thin and uniform layer on the metal surface is observed in the presence of PMBH, the cracks in the film is due to the dehydration of surface since the surface was dried prior the SEM imaging. This is evidence that PMBH can be absorbed on the mild steel surface and insulate the surface from the acidic medium.

## 3. Experimental

### 3.1. Synthesis

All of the chemicals used in this synthesis were of reagent grade (supplied by Sigma-Aldrich, Selangor, Malaysia) and were used as received without further purification. Fourier transform infrared (FT-IR) spectra were recorded using a Thermo Scientific Nicolate 6700 FT-IR Spectrometer (Thermo Fisher Scientific, Waltham, MA, USA). Nuclear magnetic resonance (NMR) spectra were recorded using an AVANCE III 600 MHz spectrometer (Bruker, Billerica, MA, USA).

#### 3.1.1. Synthesis of Thiosemicarbazone **1**

A solution of thiosemicarbazide (0.8 mM) in ethanol 100 mL was refluxed with terephthalaldehyde (0.4 mmol) for 4 h. A few drops of hydrochloric acid were added as a catalyst. The mixture was left to react for an additional 6 h to form thiosemicarbazone. After cooling to room temperature, a solid mass separated and recrystallized from ethanol; there was an 89% yield.

#### 3.1.2. Synthesis of PMBH *N*,*N*′-((2E,2′E)-2,2′-(1,4-phenylenebis(methanylylidene))bis (hydrazinecarbonothioyl))bis(2-oxo-2H-chromene-3-carboxamide) **2**

Thiosemicarbazone (1.0 mmol) in ethanol 25 mL was refluxed with coumarin-3-carboxylic acid (2.0 mmol) for 8 h. After concentrating the reaction mixture, a solid mass separated out and was recrystallized using ethanol; there was a 43% yield. The final product was then analyzed by Proton-NMR (^1^H-NMR), Carbon-13 NMR (^13^C-NMR) and FT-IR. The analysis results can be found in the supplementary files.

### 3.2. Electrochemical Measurements

Mild steel specimens obtained from the Metal Samples Company were used as the working electrodes throughout this study with active surface area of 4.5 cm^2^. The composition (wt%) of the mild steel was as follows: Fe, 99.21; C, 0.21; Si, 0.38; P, 0.09; S, 0.05; Mn, 0.05; and Al, 0.01. The specimens were cleaned according to the ASTM standard procedure G1-03 [31]. The measurements were conducted in aerated, non-stirred 1.0 M HCl solutions containing different concentrations of PMBH as inhibitor. Electrochemical measurements were performed at the steady-state corrosion potential using a Gamry water-jacketed glass cell. The cell contains three electrodes: working, counter and reference electrodes, which were composed of mild steel, a graphite bar and a saturated calomel electrode (SCE), respectively. The measurements were performed using the Gamry Instrument Potentiostat/Galvanostat/ZRA (REF 600) model (Gamry, Warminster, PA, USA). DC105 and EIS300 software by Gamry were used to perform the corrosion potential, potentiodynamic polarization, electrochemical impedance spectroscopy (EIS) and Electrochemical frequency modulation (EFM) measurements. The potentiodynamic polarization curves were swept from −0.2 to +0.2 V_SCE_ over the corrosion potential at a scan rate of 0.5 mV·s^−1^. The EIS measurements were performed using the AC signals of the 5 mV peak-to-peak amplitude at the corrosion potential in the frequency range of 100 KHz to 0.1 Hz. All of the impedance data were fit to appropriate equivalent circuits (ECs) using the Gamry Echem Analyst software. The EFM measurements were carried out at 0.1 Hz base frequency with applied AC potential of 10 mV for 20 cycles. The electrochemical measurements began to be collected approximately 30 min after the working electrode was immersed in the solution to allow the steady-state potential to stabilize. Each measurement was repeated three times, and only the average values were reported to verify the reproducibility of the experiments.

### 3.3. Surface Analysis

The surface morphology of the corroded samples was examined by scanning electron microscopy (SEM, TM1000 Hitachi Tabletop Microscope, Hitachi, Krefeld, Germany). The mild steel samples were immersed in HCl, both with and without the corrosion inhibitor, for 3 h. After the immersion, the samples were rinsed with distillated water and air dried before imaging the samples by SEM.

## 4. Conclusions

In this study, a new coumarin derivative, PMBH, was successively synthesized and characterized using various spectroscopic methods. Changes in the corrosion potential, potentiodynamic polarization, electrochemical impedance spectroscopy (EIS), and electrochemical frequency modulation (EFM) were used to study the inhibitory effect of PMBH on the corrosion of mild steel in 1.0 M HCl solution. This compound exhibited excellent inhibition performance as a mixed-type inhibitor. In general, the acidic corrosion of mild steel was reduced upon the addition of an appropriate concentration of PMBH. The inhibition efficiencies increased with inhibitor concentration but were reduced proportionally with temperature. The inhibition efficiencies obtained from various electrochemical measurements were comparable to each other. PMBH act as efficient corrosion inhibitors in 1.0 M HCl and it exhibit a maximum inhibition efficiency of 97%. SEM micrographs showed that the inhibitor molecule form a good protective film on the steel surface.

## References

[1] Rani B.E.A., Basu B.B.J. (2012). Green inhibitors for corrosion protection of metals and alloys: An overview. Int. J. Corros..

[2] Shukla S.K., Singh A., Quraishi A. (2012). Triazines: Efficient corrosion inhibitors for mild steel in hydrochloric acid solution. Int. J. Electrochem. Sci..

[3] Bin X., Wenzhong Y., Ying L., Xiaoshuang Y., Weinan G., Yizhong C. (2014). Experimental and theoretical evaluation of two pyridinecarboxaldehyde thiosemicarbazone compounds as corrosion inhibitors for mild steel in hydrochloric acid solution. Corros. Sci..

[4] Kosari A., Moayed M.H., Davoodi A., Parvizi R., Momeni M., Eshghi H., Moradi H. (2014). Electrochemical and quantum chemical assessment of two organic compounds from pyridine derivatives as corrosion inhibitors for mild steel in HCl solution under stagnant condition and hydrodynamic flow. Corros. Sci..

[5] Bobina M., Kellenberger A., Millet J., Muntean C., Vaszilcsin N. (2013). Corrosion resistance of carbon steel in weak acid solutions in the presence of L-histidine as corrosion inhibitor. Corros. Sci..

[6] Fragoza-Mar L., Olivares-Xometl O., Domínguez-Aguilar M., Flores E., Lozada P., Jiménez-Cruz F. (2012). Corrosion inhibitor activity of 1,3-diketone malonates for mild steel in aqueous hydrochloric acid solution. Corros. Sci..

[7] Hosseini M., Mertens S.F.L., Ghorbani M., Arshadi M.R. (2003). Asymmetrical Schiff bases as inhibitors of mild steel corrosion in sulphuric acid media. Mater. Chem. Phys..

[8] Yohai L., Vázquez M., Valcarce M.B. (2013). Phosphate ions as corrosion inhibitors for reinforcement steel in chloride-rich environments. Electrochim. Acta.

[9] Saravanamoorthy S., Velmathi S. (2013). Physiochemical interactions of chiral Schiff bases on high carbon steel surface: Corrosion inhibition in acidic media. Prog. Org. Coat..

[10] Nam N.D., Bui Q.V., Mathesh M., Tan M.Y.J., Forsyth M. (2013). A study of 4-carboxyphenylboronic acid as a corrosion inhibitor for steel in carbon dioxide containing environments. Corros. Sci..

[11] Sing D.N., Dey A.K. (2007). Localized coating failure of epoxy-coated Aluminium alloy 2024-T3 in 0.5 M NaCl solutions: Correlation between coating degradation, blister formation and local chemistry within blisters. Corros. Sci..

[12] Banerjee G., Mahotra S.N. (1992). Contribution to adsorption of aromatic amines on mild steel surface from HCl solutions by impedance, UV, and Raman spectroscopy. Corrosion.

[13] Arab S.T., Noor E.A. (1993). Inhibition of acid corrosion of steel by some S-alkylisothiouronium iodides. Corrosion.

[14] Hegazy M.A., Zaky M.F. (2010). Inhibition effect of novel nonionic surfactants on the corrosion of carbon steel in acidic medium. Corros. Sci..

[15] Xianghong L., Guannan M. (2005). Tween-40 as corrosion inhibitor for cold rolled steel in sulphuric acid: Weight loss study, electrochemical characterization, and AFM. Appl. Surf. Sci..

[16] Scendo M., Uznanska J. (2011). Inhibition effect of 1-butyl-4-methylpyridinium tetrafluoroborate on the corrosion of copper in phosphate solutions. Int. J. Corros..

[17] Eddy N.O., Ebenso E.E. (2008). Adsorption and inhibitive properties of ethanol extracts of Musa sapientum peels as a green corrosion inhibitor for mild steel in H_2_SO_4_. Afr. J. Pure Appl. Chem..

[18] Laamari M.R., Benzakour J., Berrekhis F., Bakasse M., Villemin D. (2012). Adsorption and kinetic studies of piperidin-1-yl-phosphonic acid as a corrosion inhibitor of iron in sulphuric acid medium. J. Mater. Environ. Sci..

[19] Wang L. (2001). Inhibiting effect of 2-mercaptopyrimidine on the corrosion of a low carbon steel in phosphoric acid. Corros. Sci..

[20] Sekine I., Nakahata Y., Tanabe H. (1988). The corrosion inhibition of mild steel by ascorbic and folic acids. Corros. Sci..

[21] Fengling X.U., Baorong H.O.U. (2009). Triazole derivatives as corrosion inhibitors for mild steel in hydrochloric acid solution. Acta Metall. Sin..

[22] Manahan S.E. (1994). Environmental Chemistry.

[23] Popova A., Christov M. (2006). Evaluation of impedance measurements on mild steel corrosion in acid media in the presence of heterocyclic compounds. Corros. Sci..

[24] Liu F.G., Du M., Zhang J., Qiu M. (2009). Electrochemical behavior of Q235 steel in saltwater saturated with carbon dioxide based on new imidazoline derivative inhibitor. Corros. Sci..

[25] Li W.H., He Q., Pei C.L., Hou B.R. (2008). Some new triazole derivatives as inhibitors for mild steel corrosion in acidic medium. J. Appl. Electrochem..

[26] Musa A.Y., Mohamad A.B., Kadhum A.A.H., Takriff M.S. (2012). Corrosion inhibition of mild steel in 1.0 M HCL by amino compound: Electrochemical and DFT studies. Metall. Mater. Trans. A.

[27] Sorkhabi H., Asghari E., Ejbari P. (2011). Electrochemical studies of adsorption and inhibitive performance of basic yellow 28 dye on mild steel corrosion in acid solutions. Acta Chim. Slov..

[28] Soltani N., Tavakkoli N., Khayatkashani M., Jalali M.R., Mosavizade A. (2012). Green approach to corrosion inhibition of 304 stainless steel in hydrochloric acid solution by the extract of Salvia officinalis leaves. Corros. Sci..

[29] De-Souza F.S. (2009). Caffeic acid as a green corrosion inhibitor for mild steel. Corros. Sci..

[30] Hermas A.A., Morad M.S. (2008). A comparative study on the corrosion behaviour of 304 austenitic stainless steel in sulfamic and sulfuric acid solutions. Corros. Sci..

[31] (2003). ASTM G1-03. Standard Practice for Preparing, Cleaning, and Evaluating Corrosion Test Specimens.

